# Breaking the silence: employees’ experiences of workplace mistreatment

**DOI:** 10.1186/s40359-026-05189-8

**Published:** 2026-07-21

**Authors:** Mohamed Mohiya

**Affiliations:** Department of Human Resources Management, King Khalid University, Abha, Saudi Arabia

**Keywords:** Unacceptable workplace behavior, Organizational justice, Psychological safety, Employee voice, Organizational silence, Workplace mistreatment, Saudi Arabia, Qualitative research, Thematic analysis

## Abstract

**Background:**

Harmful workplace conduct—verbal aggression, authority misuse, and discriminatory practices—compromises employee well-being, organizational fairness, and psychological safety. Theoretically grounded qualitative research in non-Western, collectivist settings remains scarce. This single-case, exploratory qualitative study examines how employees at a large Saudi multinational experience unacceptable workplace behavior, focusing on organizational justice, psychological safety, and employee voice. Drawing on Organizational Justice Theory and Psychological Safety Theory, the study frames misconduct as justice violations that erode safety conditions and suppress voice.

**Methods:**

This single-case exploratory study triangulated two evidence sources: (1) thematic analysis of 222 employee-generated comments from the organization’s internal social media (collected in 2014), and (2) 39 semi-structured interviews across job levels, functions, and nationalities (2017–2020). The temporal gap between sources reflects the inquiry’s longitudinal depth; the interview phase explicitly examined whether patterns observed in earlier comments persisted over time.

**Results:**

The analysis produced five principal themes: (1) widespread power imbalances and authority misuse; (2) organizational silence driven by fear of reprisal; (3) entrenched violations of organizational justice; (4) breaches of the psychological contract coupled with institutional distrust; and (5) cultural and situational dynamics influenced by hierarchical norms, collectivist values, and religious principles.

**Conclusion:**

The study advances theory through the Justice–Safety Vicious Cycle Model—an interpretive framework grounded in participants’ accounts—demonstrating the reciprocal, self-reinforcing link between justice violations and the erosion of psychological safety. Empirically, it offers rare qualitative insight into Saudi organizational conduct, including the novel phenomenon of weaponizing performance management. Practically, it provides evidence-based guidance for culturally attuned interventions to restore psychological safety and reinforce leadership accountability.

## Introduction and research background

### Background

Modern organizations increasingly recognize that ethical, respectful, and psychologically safe workplaces are fundamental to achieving sustained competitive advantage, promoting employee well-being, and enhancing overall organizational performance [26, 73]. The manner in which employees are treated on an interpersonal level profoundly affects their job satisfaction, organizational commitment, work performance, and mental health [23]. In contrast, unacceptable workplace behaviors—spanning verbal abuse, exploitation of positional authority, discriminatory conduct, bullying, and deliberate exclusion—impose significant burdens on individuals, workgroups, and organizations as a whole [45, 70].

Meta-analytical findings have consistently highlighted the serious adverse consequences associated with workplace mistreatment. Workers exposed to abusive supervisory conduct, incivility, or discriminatory treatment demonstrate increased emotional exhaustion, elevated psychological distress, lower job satisfaction, reduced organizational commitment, and heightened intentions to leave [58, 78]. Furthermore, witnessing mistreatment directed at coworkers can itself produce secondary trauma and erode shared perceptions of psychological safety within teams [88]. At the organizational level, such experiences translate into measurable losses in productivity, elevated turnover costs, heightened legal exposure, and reputational harm [73].

Despite this well-established body of evidence, scholarly insight into how employees actually experience, make sense of, and respond to unacceptable conduct remains underdeveloped—particularly in non-Western organizational contexts characterized by distinct cultural traditions, power arrangements, and social expectations [5, 74]. The broader Middle East region, and Saudi Arabia in particular, constitutes a significantly underexplored territory within the organizational behavior literature [59].

### Research problem and major gaps

Despite growing scholarly interest in workplace ethics, several important knowledge gaps persist. To begin with, most investigations into unacceptable workplace behavior depend on quantitative research designs that provide limited access to the subjective experiences, interpretive processes, and contextual intricacies of those affected [42]. Qualitative investigations that capture the lived dimensions of workplace mistreatment remain relatively uncommon [57].

Second, theory-informed qualitative studies that deliberately situate employees’ experiences within recognized theoretical frameworks are largely missing from the literature. A great deal of qualitative research approaches the topic descriptively, without systematic grounding in established theory, which limits the potential for meaningful theoretical advancement [87]. Third, the preponderance of workplace behavior scholarship has been conducted in Western settings [26], leaving the Middle Eastern context severely underrepresented [18].

Fourth, prior research has not adequately explored the mediating roles of organizational silence and psychological safety in the relationship between unacceptable conduct and employee outcomes [62, 68]. Finally, scholarly attention to how differences in employment status interact with experiences of mistreatment and perceptions of justice has been limited [54].

### Theoretical foundation

To address these deficits, the present study adopts an integrative theoretical framework drawing on Organizational Justice Theory [21, 44] and Psychological Safety Theory [32, 49]. This combination is particularly well suited to the present inquiry, as the data indicate that employees’ experiences inherently entail both fairness evaluations and concerns about safety and voice.

Organizational Justice Theory offers a comprehensive lens for examining how employees appraise the fairness of outcomes (distributive justice), decision-making processes (procedural justice), and the quality of interpersonal treatment they receive (interactional justice) [22]. Unacceptable workplace behaviors represent violations of interactional justice insofar as they involve treatment that fails to uphold standards of dignity, respect, and propriety [11]. When organizations neglect to institute clear, equitable processes for managing misconduct, violations of procedural justice are layered atop these interactional transgressions [56].

Psychological Safety Theory examines how employees perceive the consequences of engaging in interpersonal risk-taking within their work settings [32]. When psychological safety is present, employees feel free to raise concerns without dreading negative outcomes [49]. Conversely, when employees believe that voicing concerns could invite retaliation, psychological safety deteriorates, and organizational silence takes hold [63].

The integration of these two frameworks is mutually reinforcing: justice violations directly compromise psychological safety by communicating that the environment punishes rather than shields those who challenge misconduct [66]. At the same time, the absence of psychological safety sustains justice violations by discouraging victims from reporting mistreatment [30]. This bidirectional dynamic generates self-reinforcing cycles in which initial justice transgressions suppress voice, thereby permitting continued violations that further undermine perceptions of fairness and safety.

#### Research aim

This investigation seeks to develop a rich, context-sensitive, and theoretically grounded understanding of how workers within a large Saudi multinational corporation encounter, interpret, and navigate unacceptable workplace behaviors, with a focus on their perceptions of organizational justice, psychological safety, and the dynamics of employee voice.

#### Research objectives

- To identify and describe the specific forms of unacceptable workplace conduct that employees encounter.
- To examine how employees interpret such conduct through the lens of organizational justice.
- To explore the connections between experiences of unacceptable behavior and perceptions of psychological safety.
- To investigate the mechanisms through which organizational silence takes root and becomes entrenched.
- To analyze how contextual factors shape employees’ experiences of and reactions to unacceptable conduct.

#### Research questions

- RQ1: Which forms of unacceptable workplace conduct do employees consider most problematic, and how do they describe its consequences?
- RQ2: In what ways do employees’ encounters with unacceptable behavior relate to their perceptions of organizational justice?
- RQ3: What is the nature of the relationship among unacceptable workplace behavior, psychological safety, and employee voice?
- RQ4: In what ways do organizational systems, structures, and policies either enable or sustain unacceptable behaviors?
- RQ5: How do cultural norms, hierarchical arrangements, and employment status categories intersect with employees’ experiences of and reactions to unacceptable conduct?

This research makes a significant contribution as one of the earliest theory-driven qualitative examinations of workplace behavior in Saudi organizational contexts. It extends justice and safety theories by clarifying their applicability in non-Western, hierarchical, and collectivist settings, yields practical insights for organizations, and demonstrates methodological innovation by combining organically occurring organizational discourse with conventional interview-based data.

## Literature review

### Conceptualization and theoretical foundation

#### Defining unacceptable workplace behavior

Unacceptable workplace behavior refers to any harmful conduct that transgresses organizational norms, societal standards of civil treatment, and core principles of human dignity [72]. Across the scholarly literature, this phenomenon has been examined under various labels—including workplace aggression [67], incivility [4], abusive supervision [83], and bullying [36]—yet all share a common thread: actions that harm or carry the potential to harm employees’ well-being, dignity, or professional standing.

For the present investigation, an inclusive working definition is adopted, whereby unacceptable workplace behavior encompasses any conduct that: (1) contravenes organizational values centered on dignity and respect; (2) generates environments marked by hostility, intimidation, or denigration; (3) exploits formal authority; (4) discriminates against individuals on protected grounds; or (5) deliberately suppresses employee voice. This definition extends to both high-intensity behaviors such as verbal abuse and direct threats, as well as lower-intensity but cumulatively harmful behaviors such as chronic dismissiveness and social exclusion.

#### Organizational justice theory

Organizational Justice Theory provides an integrative conceptual lens for understanding how employees respond to the treatment they receive in the workplace [21, 44]. Central to the theory is the premise that employees continuously evaluate the fairness of their organizational experiences across several dimensions, and that these evaluations exert substantial influence on their attitudes, emotional states, and workplace conduct [20].

Distributive Justice concerns employees’ appraisals of the fairness of outcome allocations—encompassing rewards, resources, opportunities, and sanctions [1]. Employees assess whether the outcomes they receive align with applicable allocation norms and are treated equitably relative to relevant comparison groups [22].

Procedural Justice pertains to perceived fairness in the processes used to reach decisions [53, 86]. According to Leventhal’s [53] framework, fair procedures must be applied consistently, free from bias, grounded in accurate information, open to correction, inclusive of relevant voices, and ethically defensible. In the context of unacceptable workplace behavior, procedural justice concerns frequently arise regarding complaint investigation mechanisms and accountability systems [37].

Interactional Justice refers to the quality of interpersonal treatment that individuals receive [11], and is further differentiated into interpersonal justice (the extent to which individuals are treated with dignity, courtesy, and respect) and informational justice (the adequacy and honesty of explanations provided) [21]. At their core, unacceptable workplace behaviors constitute interactional injustice—conduct that violates established norms of dignified treatment [10].

Meta-analytical research consistently demonstrates that justice perceptions predict a wide range of outcomes, including organizational commitment, trust, job performance, withdrawal behavior, and psychological well-being [23]. Interactional justice is particularly strongly associated with supervisor-related outcomes, while procedural justice tends to predict organization-directed responses—a pattern known as the “target similarity effect” [76].

#### Psychological safety theory

Psychological Safety is conceptualized as “a shared belief that the team is safe for interpersonal risk taking” ([32], p. 350). When this condition exists, individuals feel at liberty to express themselves openly, raise concerns, and acknowledge mistakes without fear of harm to their image, status, or career trajectory [49]. When psychological safety is absent, organizational silence tends to emerge, with employees choosing to withhold information and concerns rather than risk-averse consequences [63].

Edmondson’s [32] research established that psychological safety functions as both a team-level and an organizational-level construct, shaped by leaders’ behavior, organizational policies and practices, group norms, and the accumulated history of rewarding or penalizing employees who speak up. Leaders foster psychological safety through their approachability, active solicitation of input, acknowledgment of their own limitations, and willingness to receive feedback without becoming defensive [33].

Employee Voice—described as “discretionary communication of ideas, suggestions, concerns, or opinions about work-related issues with intent to bring about improvement” ([61], p. 375)—constitutes a central expression of psychological safety. Voice behaviors facilitate error identification, problem recognition, and shared organizational learning [30]. However, speaking up carries interpersonal risks: potential embarrassment, strained relationships, or career-related setbacks [60].

A consistent body of research identifies fear as the primary obstacle to employee voice [62]. Employees often remain silent when they anticipate that speaking up will invite negative responses—such as being labeled as troublemakers, damaging their standing with supervisors, or facing retaliatory treatment [60]. These concerns are especially pronounced when the issue at hand involves misconduct by those holding organizational power [31].

Organizational Silence: Building on prior work, Morrison and Milliken [63] distinguished between individually motivated silence and systemic organizational silence, which emerges when collective beliefs take hold that voicing concerns is either pointless or hazardous. This systemic form of silence carries serious organizational consequences: problems go undetected, employee cynicism deepens, innovation is stifled, and justice violations continue without correction [90].

#### Theoretical integration

The relationship between organizational justice and psychological safety is characterized by bidirectionality and mutual reinforcement [80]. Violations of justice—especially procedural and interactional dimensions—erode psychological safety by signaling that the environment penalizes those who raise concerns [66]. Concretely, when complaint processes operate inconsistently or retaliate against reporters, they signal that voicing is futile and hazardous, directly degrading employees’ perceptions of safety [30]. Conversely, when psychological safety is absent, employees cannot engage in the corrective voice behavior needed to address justice violations [82]; silence becomes the rational response, allowing misconduct to continue unchallenged. This dynamic generates virtuous cycles (justice → safety → voice → sustained justice) or vicious ones (injustice → fear → silence → continued injustice) [19]. In hierarchical, collectivist settings such as Saudi Arabia, these feedback loops are amplified by high power-distance norms that normalize deference to authority [17] and collectivist orientations that intensify concerns about relational disruption [40], making it especially difficult to escape vicious cycles without structural intervention.

### Relevant research

#### Forms and impacts of workplace mistreatment

Empirical scholarship has extensively documented the prevalence of workplace mistreatment across organizational settings. Research estimates that abusive supervision affects roughly 13.6% of the U.S. workforce [77], while the prevalence of workplace incivility has been estimated to range from 75 to 98% [24]. Commonly identified manifestations include verbal aggression [67], abusive supervision [84], workplace bullying [36], and discrimination [43].

The consequences for those affected are well established. Victims frequently experience heightened psychological distress, depression, anxiety, emotional exhaustion, and deteriorating physical health [12, 69]. In terms of occupational outcomes, mistreatment is associated with reduced job satisfaction, weakened organizational commitment, lower performance, and increased intentions to quit [46].

#### Power, hierarchy, and supervisory mistreatment

Research consistently identifies power differentials as a central feature of mistreatment dynamics. Those who occupy positions of formal authority are better positioned to harm subordinates while simultaneously facing less organizational accountability for doing so [85]. The extent of supervisors’ control over pivotal career outcomes—including performance appraisals, task allocations, and access to developmental opportunities—amplifies the vulnerability of those in subordinate roles and limits their ability to respond [84].

#### Voice, silence, and reporting barriers

Cortina and Magley’s [25] research revealed that only around 30% of harassment victims pursue formal complaints. Fear of retaliation stands out as the principal deterrent [65]. Workers anticipate detrimental consequences for their careers—including unfavorable performance ratings, missed promotion opportunities, and even termination—especially when those responsible for the misconduct hold significant organizational power [60].

A further impediment is the anticipation of futility: many employees choose to remain silent because they believe that reporting misconduct will produce no meaningful change or that their concerns will not be taken seriously [62]. Concerns about relationship damage are particularly salient in collectivist organizational cultures, where raising formal complaints can be seen as disruptive and may undermine group cohesion [61].

#### Cultural context and workplace behavior

Cross-cultural research has revealed that norms governing workplace behavior and the strategies employees adopt in response to mistreatment vary considerably across cultural settings [5]. Power distance—defined as the degree to which individuals with less power in a society accept and expect unequal distributions of power [47]—is especially relevant. Cultures characterized by high power distance tend to exhibit greater tolerance for autocratic management styles and hierarchically structured decision-making [74].

The collectivism-individualism dimension shapes whether employees give greater weight to personal rights or group harmony when deciding how to respond to mistreatment [40]. Collectivist orientations that prioritize maintaining relationships and avoiding conflict may heighten employees’ reluctance to report misconduct [61].

Despite the region’s economic significance, organizational behavior research in Middle Eastern contexts remains strikingly sparse [59]. Islamic values centered on human dignity (karama) and justice (adl) may provide strong normative foundations for condemning mistreatment in these settings [7].

#### Workplace mistreatment in the middle east and GCC context

Despite the economic significance of GCC states, organizational behavior scholarship in this region remains limited [59]. Available studies highlight high power distance, paternalistic leadership, and in-group favoritism (wasta) as salient features of Saudi organizational life [5, 74]. Saudi Arabia’s rapid modernization under Vision 2030 has created tensions between traditional hierarchical norms and emerging expectations for transparency and employee voice, making this context theoretically significant for understanding how workplace mistreatment is experienced in non-Western settings. The present study directly addresses this documented gap. An emerging body of scholarship examines how workers’ employment classification shapes their organizational experiences [54]. Non-permanent workers frequently encounter what amounts to second-class treatment, including differential handling, social marginalization, and constrained access to organizational resources [91]. Employment status may intersect with vulnerability to unacceptable conduct, as contractors face compounded risks stemming from job insecurity and diminished organizational voice [39].

## Methodology

### Research design and philosophical positioning

The present investigation employs a qualitative, exploratory, theory-informed single-case study design [93] to examine workers’ experiences of unacceptable workplace conduct within a large Saudi-based multinational organization. The case study methodology is well suited to inquiries into complex, context-dependent phenomena [81]. The single-case design is justified on three grounds: (1) the organization represents a revelatory case in Yin’s [93] sense, providing rare access to a phenomenon previously inaccessible to researchers; (2) its combination of scale, workforce diversity, explicit misconduct policies, and GCC context makes it theoretically representative of large Middle Eastern multinationals; and (3) depth of evidence from two complementary sources enables nuanced analysis not achievable through multi-case snapshot designs.

Philosophical Positioning: The study is anchored in a social constructivist epistemological orientation [8], which holds that individuals actively construct meaning through engagement with the world and that multiple, socially situated realities emerge from diverse standpoints [55]. Alongside this, the study maintains a critical realist ontological position [9], acknowledging that while individual interpretations differ, underlying structural forces—such as power asymmetries, organizational arrangements, and cultural frameworks—exist independently of individuals’ perceptions and exert genuine causal influence [28].

### Research context

The research was conducted within a large multinational organization based in Saudi Arabia and operating in the energy sector, with a workforce exceeding 60,000 employees. The organization has a substantial international footprint and employs a highly diverse workforce comprising Saudi nationals, Arab expatriates, and professionals of Western and Asian backgrounds. It is recognized for its professionally oriented management practices and its stated commitment to core organizational values, including integrity, excellence, and respect.

Rationale for Case Selection: The organization was chosen through purposive sampling based on several criteria: (1) its scale and organizational complexity, which generate a broad range of workplace behavior experiences; (2) its workforce diversity, which enables examination of how nationality, employment classification, and cultural background intersect with mistreatment experiences; (3) the existence of formal policies expressly prohibiting workplace misconduct; (4) the accessibility of data; and (5) its representativeness of large Middle Eastern organizations currently undergoing processes of modernization.

### Data sources

#### Content analysis: employee comments from internal social media

The principal data source consists of 222 employee-generated comments extracted and analyzed through content analysis from the organization’s internal social media, specifically a post titled “Unacceptable Behavior in the Workplace.” The internal social media platform, hosted on the organization’s intranet and available to all staff, functions as a channel for HR communications and employee engagement. The post in question specifically invited employees to share their experiences, concerns, and recommendations regarding workplace conduct.

These organically occurring data carry distinct advantages over researcher-elicited material. They reflect employees’ unprompted, self-initiated concerns and are not influenced by interviewer cues or tendencies toward socially acceptable responses. As such, they represent an authentic form of organizational discourse that reveals the language, framings, and concerns employees naturally draw upon when communicating within their own professional community [51]. The comments examined in this study were submitted over six months in 2014. Although these data predate the interview phase (2017–2020), the temporal gap was treated as an analytical resource: the interview guide explicitly asked participants whether the conduct patterns they described had changed in recent years, enabling cross-temporal comparison. The consistent convergence of findings across both sources suggests that the identified dynamics reflect durable organizational patterns rather than momentary occurrences.

#### Semi-structured interviews

The secondary data source comprises 39 semi-structured interviews with organizational employees conducted between 2017 and 2020. Each interview lasted between 45 and 90 min, was audio-recorded with participants’ informed consent, and was subsequently transcribed in full.

The interview guide (Appendix A) included open-ended questions designed to explore: (1) employees’ conceptual understanding of unacceptable workplace behavior; (2) personal experiences or observations of such conduct; (3) perceptions of how the organization responds; (4) the considerations shaping decisions to speak up or remain silent; (5) proposed improvements to organizational processes; and (6) reflections on how cultural factors affect these dynamics. The semi-structured format ensured consistent coverage of key topics while preserving the flexibility needed to explore emergent themes in depth [16].

### Participants and sampling

The 222 comments represent a self-selected group of contributors. Although complete demographic data are not available for all commenters, the available evidence suggests a diverse pool that includes Saudi nationals and expatriates from multiple countries, employees at various organizational levels, individuals of both genders, and workers with varying lengths of service.

Interview participants were identified through a combination of purposive and snowball sampling strategies [71] to ensure diverse representation across key characteristics (Table 1).

**Table 1:** Interview participant demographics (*N* = 39)

Characteristic	Category	*N*	%
Gender	Male	29	74.4
	Female	10	25.6
Nationality	Saudi	17	43.6
	Arab (other)	11	28.2
	Asian	7	17.9
	Western	4	10.3
Employment Status	Direct Hire	25	64.1
	Contractor	14	35.9
Level	Frontline/Technical	15	38.5
	Professional/Specialist	17	43.6
	Supervisory/Managerial	7	17.9
Tenure	< 2 years	7	17.9
	2–5 years	13	33.3
	6–10 years	11	28.2
	> 10 years	8	20.5

### Data analysis

Data analysis followed Braun and Clarke’s [13, 15] six-phase thematic analysis framework, which provides a rigorous yet adaptable process for identifying meaningful patterns across qualitative datasets. This approach accommodates both inductive coding strategies driven by the data and deductive strategies guided by theoretical frameworks [14].

#### Phase 1: familiarization

The research team immersed itself in the data through repeated, careful readings, recording initial reactions and noting emerging patterns.

#### Phase 2: generating initial codes

A systematic coding process was carried out to identify meaningful segments within the data. This involved applying both deductively derived codes grounded in theoretical constructs (such as “procedural injustice” and “fear of retaliation”) and inductively derived codes that emerged organically from the data (such as “contractor discrimination” and “PMP threats”), using NVivo 12. The primary researcher conducted the coding, which a qualitative research colleague with expertise in organizational behavior independently verified. Disagreements were resolved through discussion until consensus was reached. Thematic saturation was assessed iteratively; no new codes emerged after approximately the 30th interview, and the full comment dataset was reviewed to confirm that all coded patterns were represented across both sources.

#### Phase 3: searching for themes

Codes were grouped into candidate themes that captured broader patterns of shared meaning across the data.

#### Phase 4: reviewing themes

Candidate themes were subjected to iterative review to confirm internal coherence and meaningful differentiation from one another [71].

#### Phase 5: defining and naming themes

Each theme received a precise definition, its boundaries were clarified, and its connection to the research questions was made explicit.

#### Phase 6: producing the report

The final analytic report situated the thematic findings within the relevant theoretical frameworks and incorporated illustrative quotations.

### Rigor and trustworthiness

Credibility was supported through methodological triangulation, sustained engagement with the field, member checking, and peer debriefing. Transferability was promoted through detailed contextual description, strategic sampling, and theoretical grounding. Dependability was established through maintaining an audit trail, adhering to systematic analytic procedures, and involving multiple coders. Confirmability was upheld through reflexive practice, transparent reporting, and a firm grounding of all interpretations in the data [55].

#### Researcher positionality

As a Saudi national with professional experience in Saudi organizations, the researcher brought contextual familiarity that enhanced interpretive sensitivity to cultural and hierarchical nuances. To guard against confirmatory bias, reflexive memos were maintained throughout analysis, interpretations were shared with participants for member checking, and a research colleague external to the Saudi organizational context provided critical peer debriefing at key analytical junctures [38].

### Ethical considerations

The study received formal approval from the relevant Institutional Review Board. Interview participants were provided with comprehensive information about the study prior to their involvement and gave written informed consent. The content analysis of comments from the internal social media constituted a secondary analysis of data hosted by the organization; nevertheless, all comments were fully anonymized, and any potentially identifying details were removed. All data were stored in password-protected files accessible only to members of the research team.

## Findings

Thematic analysis produced five overarching themes that together capture how employees experience unacceptable workplace conduct and how these experiences intersect with organizational justice, psychological safety, and voice (Table 2).

**Table 2:** Themes, subthemes, and prevalence

Theme	Sub-themes	% of Coded Segments (NVivo references)	Sources
Theme 1: Pervasive Power Asymmetries and Abuse of Authority	1.1 Verbal Aggression and Hostile Communication 1.2 Threats and Intimidation 1.3 Post-Promotion Arrogance 1.4 Weaponization of Performance Management	35%	Internal Social Media: 127 comments Interviews: 38
Theme 2: Organizational Silence and Fear of Retaliation	2.1 Career Consequences and PMP Manipulation 2.2 “Creating Enemies” in the Workplace 2.3 Complaints Never Reach Anywhere 2.4 Institutional Distrust	28%	Internal Social Media: 98 comments Interviews: 40
Theme 3: Systemic Justice Violations	3.1 Contractor vs. Direct-Hire Discrimination 3.2 Favoritism and “Wasta” 3.3 Inequitable Policy Application 3.4 Procedural Inconsistencies	18%	Internal Social Media: 73 comments Interviews: 35
Theme 4: Psychological Contract Breaches and Institutional Distrust	4.1 Gap Between Policy and Practice 4.2 Zero Tolerance Not Enforced 4.3 Perpetrators Protected, Victims Punished 4.4 Organizational Cynicism	12%	Internal Social Media: 54 comments Interviews: 29
Theme 5: Cultural and Contextual Dynamics	5.1 Respect for Hierarchy vs. Dignity Violations 5.2 Collectivism and Harmony Preservation 5.3 Islamic Values and Behavioral Expectations 5.4 Generational Divides	7%	Internal Social Media: 38 comments Interviews: 24

### Theme 1: pervasive power asymmetries and abuse of authority (35%)

The most dominant theme in participants’ accounts was perceived misuse of systematic supervisory power, manifesting as verbal aggression, intimidation, and what many described as manipulation of performance appraisal systems. As reported by participants, these behaviors constitute violations of interactional justice, compounded by entrenched power imbalances.

#### Verbal aggression and hostile communication

Shouting, yelling, profane language, and aggressive interpersonal communication were the most frequently cited behaviors across data sources:

*“Always the Managers utilize bad words and swear at employees in a bad manner and utilize their position of authority to threaten their subordinates… we can not report it since we do not want to create enemies in the workplace.” (Internal Social Media Comment)*.

*“When my supervisor shouts at me in front of others*,* I feel so small*,* so disrespected. It is not just about the words—it is about making you feel worthless. Moreover*,* you cannot say anything back because he controls your PMP*,* your future here.” (Interview P-23)*.

These encounters represent unambiguous interactional injustice—treatment that fundamentally disregards employees’ dignity and respect [11]. The public dimension of such conduct exacerbates its impact by introducing elements of status degradation [10].

#### Threats and intimidation

Participants described explicit coercive threats that linked compliance with employment security and career advancement:

*“My supervisor made it very clear—if I do not agree to work overtime every day*,* including weekends*,* he will give me a poor rating. He said directly*,* ‘Your PMP depends on your cooperation.’” (Interview P-31)*.

Such threats constitute serious violations of interactional justice while simultaneously undermining psychological safety by signaling to employees that the environment punishes rather than protects interpersonal risk-taking [32].

#### Post-promotion arrogance

A recurring observation concerned the behavioral changes that followed supervisory promotions:

*“I worked with this person for three years as colleagues. We were friends. Then he got promoted to supervisor*,* and suddenly he treats me like I am nobody—ignoring my questions*,* criticizing my work publicly*,* taking credit for my ideas.” (Interview P-12)*.

This pattern points to how organizational power structures, when combined with insufficient leadership preparation, can fundamentally alter individual conduct—reflecting how hierarchical systems lacking adequate accountability mechanisms create conditions conducive to supervisory aggression [50].

#### Weaponization of performance management

Employees described the deliberate exploitation of performance management processes as instruments of retaliation:

*“If they raise a complaint*,* the retaliation is always in the form of a low PMP rating. In addition*,* no clear actions are taken against the harasser*,* which gives them ample opportunity to continue their inappropriate actions.” (Internal Social Media Comment)*.

*“Your entire career depends on your PMP rating—promotions*,* bonuses*,* training opportunities*,* everything. So when your supervisor threatens your rating*,* he has complete power over you. I have seen people accept any abuse*,* work any hours*,* stay silent about any problem*,* because they cannot risk a poor rating.” (Interview P-19)*.

The weaponization of performance management constitutes simultaneous violations of both procedural justice (performance evaluation functioning in an unfair and biased manner) and distributive justice (inequitable allocation of rewards and penalties) [21], thereby generating powerful mechanisms that suppress employee voice [63].

### Theme 2: organizational silence and fear of retaliation (28%)

The second major theme revealed a pervasive culture of fear-driven organizational silence, in which employees systematically withheld their concerns despite recognizing the harm being caused—a finding that speaks directly to the central concerns of psychological safety theory.

#### Career consequences and PMP manipulation

Fear of adverse career consequences emerged as the most powerful mechanism suppressing employee voice:

*“What if the opponent is your immediate supervisor and there are a lot of things at stake if you complain….PMP*,* promotion*,* career opportunity*,* reputation…etc.” (Internal Social Media Comment)*.

*“I witnessed my colleague report verbal abuse by our supervisor. What happened? Nothing to the supervisor. But my colleague? Suddenly*,* every small mistake was documented*,* his PMP rating dropped*,* and he was passed over for promotion. The message was clear: this is what happens when you complain. After that*,* nobody reports anything.” (Interview P-14)*.

These accounts vividly demonstrate how the erosion of psychological safety generates organizational silence [63]. Employees engage in rational cost–benefit assessments and conclude that the risks of speaking up outweigh any potential benefits [65].

#### “Creating enemies” in the workplace

Beyond formal career repercussions, employees expressed apprehension about the relational damage that reporting misconduct could entail:

*“In our culture*,* maintaining relationships is everything. When you formally complain about someone*,* especially a supervisor*,* you break that. Even if you are right*,* even if they are wrong*,* you become the problem. So people choose to keep peace rather than seek justice.” (Interview P-22)*.

The concern about “creating enemies” reflects collectivist cultural values that prioritize relationship maintenance and the preservation of group harmony [40]. In high power distance settings, openly challenging authority also transgresses powerful social norms [47], thereby compounding the psychological safety deficits already present.

#### Anticipated futility: “Complaints never reach anywhere”

Widespread belief in the futility of reporting was another prominent feature of the data:

*“The truth is there are a lot of complaints of all kinds of employee abuse. The real issue is these complaints never reach anywhere*,* and nobody observes such an attitude of harassment.” (Internal Social Media Comment)*.

*“I have seen multiple people report problems through proper channels. What happens? The complaint gets investigated by the accused person’s friends. Nothing changes. Meanwhile*,* the person who complained now has a target on their back.” (Interview P-18)*.

Perceptions of futility foster organizational cynicism [29] and reinforce silence [62], simultaneously reflecting failures of procedural justice and the erosion of psychological safety.

#### Institutional distrust

Participants conveyed a deep-seated distrust in the organization’s willingness to address abuses of power meaningfully:

*“The fundamental problem is that those with power protect each other. When you report a manager*,* the investigation is conducted by other managers. They are not going to discipline one of their own seriously. The system is designed to protect management*,* not employees.” (Interview P-24)*.

Institutional distrust reflects the cumulative weight of perceived violations of the psychological contract [75]. It fundamentally undermines psychological safety while signaling endemic failures of procedural and interactional justice throughout the organization.

### Theme 3: systemic justice violations (18%)

Beyond interpersonal mistreatment, employees identified systemic organizational practices and arrangements that themselves generate pervasive injustice.

#### Contractor vs. direct-hire discrimination

Systematic differential treatment based on employment classification was among the most frequently cited justice violations:

*“We had several experience/s with regards to the subject*,* most often with non-company employee/s. Supervisors and those in higher positions*,* in most situation/snario they*,* will treat non-company employee/s as their slaves.” (Internal Social Media Comment)*.

*“The discrimination is everywhere. Different gates for entry. Different facilities. Different treatment from supervisors. We do the same work; sometimes contractors even do more technical work*,* but we are always reminded that we are ‘just contractors.’ It creates resentment*,* and it is fundamentally unfair.” (Interview P-11)*.

*“The worst part is job insecurity. Contractors can be let go with minimal notice. So when a direct-hire supervisor abuses a contractor*,* what can they do? Report it and risk termination? The power imbalance is extreme*,* and abusive people exploit it.” (Interview P-39)*.

Employment status-based discrimination constitutes distributive injustice (unequal allocation of respect, resources, and opportunities based on categorical membership rather than merit) and interactional injustice (treatment that fails to respect workers’ dignity) [21]. This systematic stratification creates what amounts to organizational caste structures in which more vulnerable groups face heightened mistreatment risks alongside diminished recourse and voice [54].

#### Favoritism and “wasta”

Many participants identified favoritism and “wasta” (nepotism and the leveraging of personal connections) as deeply embedded justice violations:

*“Being unjust to the subordinates creates an internal society that is full of hatred and anger towards the administrations. Wasta*,* moods*,* and personal interests are ruining [the organization]” (Internal Social Media Comment)*.

*“If you have the right connections—tribal*,* family*,* nationality—you get opportunities*,* promotions*,* favorable assignments. If you do not*,* it does not matter how hard you work or how qualified you are. This breeds enormous resentment and undermines any sense that merit matters.” (Interview P-13)*.

Favoritism violates distributive justice by allocating rewards based on connections rather than equity and procedural justice by subverting principles of consistency, accuracy, and bias suppression in personnel decisions [53]. When favoritism becomes systemic, it signals that organizational systems are fundamentally unfair, cultivating widespread cynicism and eroding perceptions of collective justice [2].

#### Inequitable policy application

Participants consistently observed that policies prohibiting misconduct were not applied consistently, with enforcement standards varying according to organizational position:

*“Policies prohibit harassment*,* but when supervisors do it*,* nothing happens. When a frontline employee makes a mistake*,* there are immediate consequences. The rules do not apply equally. There is one standard for management*,* another for everyone else.” (Interview P-17)*.

Selective policy enforcement violates procedural justice principles, particularly those requiring consistency and the suppression of bias [53]. When employees perceive that rules are applied differently depending on organizational status, procedural fairness is comprehensively undermined [22].

#### Procedural inconsistencies

Employees identified widespread procedural deficits in how misconduct was handled:

*“There is no clear process. Whom do you report to? What happens then? How is confidentiality protected? What constitutes sufficient evidence? These basic questions have no clear answers*,* which makes reporting seem impossible even if you wanted to try.” (Interview P-09)*.

These procedural failings—including the absence of employee voice in processes, lack of transparency, inadequate mechanisms to suppress bias, and inconsistent application—together constitute a comprehensive failure of procedural justice [53, 86].

### Theme 4: psychological contract breaches and institutional distrust (12%)

This theme captured employees’ perceptions that the organization had failed to fulfill its commitments to dignity, respectful treatment, and fairness—representing fundamental violations of the psychological contract that engendered institutional distrust and widespread cynicism.

#### Gap between policy and practice

The most prevalent expression within this theme concerned the gulf between what the organization publicly espoused and what employees actually experienced:

*“The company has beautiful policies—respect*,* dignity*,* zero tolerance for harassment. However*,* they are just words on paper. In reality*,* abusive supervisors keep their jobs*,* favoritism continues*,* and complaints go nowhere. The gap between what is promised and what is delivered is enormous.” (Interview P-20)*.

The policy–practice gap constitutes a form of psychological contract breach, in which employees perceive that the organization has failed to honor commitments—both explicit and implicit [75]. Such violations generate feelings of betrayal, reduce organizational commitment and trust, and foster cynical attitudes [64].

#### Zero tolerance not enforced

In relation to harassment policies specifically, many employees noted that the proclaimed “zero tolerance” stance had proved to be largely illusory in practice:

*“Zero tolerance is supposed to mean exactly that—zero. One incident and you are out. But we all know supervisors who have been reported multiple times*,* and nothing happens. The policy is meaningless if there is no enforcement.” (Interview P-16)*.

Unenforced policies signal procedural injustice—demonstrating inconsistency and bias in organizational systems—and violate the psychological contract. Furthermore, the visible failure to enforce stated commitments signals that voicing concerns is futile, thereby directly eroding psychological safety [35].

#### Perpetrators protected, victims punished

Among the most damaging findings for institutional trust were accounts in which organizations actively shielded perpetrators while abandoning or retaliating against those who reported misconduct:

*“I personally know three people who reported abusive supervisors through proper channels. All three experienced retaliation—poor ratings*,* denied promotions*,* and eventual transfer to undesirable positions. The supervisors? Still in their jobs. The message could not be clearer. (Interview P-10)*

When organizations protect those responsible for misconduct while exposing those who report it to retaliation, they compound justice violations across multiple dimensions: procedural (biased investigations), distributive (perpetrators left unpunished while victims suffer), and interactional (treatment lacking basic dignity). Such patterns fundamentally destroy institutional trust and unambiguously signal the absence of psychological safety.

#### Organizational cynicism

The accumulation of psychological contract breaches and repeated justice violations gave rise to pervasive organizational cynicism:

*“I used to believe the company genuinely cared about employees. After years of witnessing the gap between policy and reality*,* I am cynical. This blog*,* these surveys*,* these initiatives—they are public relations. Real change? I do not expect it.” (Interview P-21)*.

Organizational cynicism emerges from repeated encounters with injustice, unmet commitments, and perceived institutional hypocrisy [48]. Its consequences are especially corrosive: cynical employees exhibit reduced engagement, diminished organizational citizenship behavior, and decreased willingness to provide upward feedback or participate in organizational improvement efforts [3].

### Theme 5: cultural and contextual dynamics (7%)

The final theme captured how cultural values, religious frameworks, and contextual factors shaped both the norms employees held regarding appropriate conduct and their responses when those norms were violated.

#### Respect for hierarchy vs. dignity violations

Participants articulated a tension between culturally embedded norms of deference to authority and universal principles of human dignity:

*“In our culture*,* we are taught to respect elders and those in authority. This is good. Nevertheless*,* some supervisors exploit this—they expect absolute obedience and interpret any question as disrespect. There is a difference between cultural respect and accepting abuse.” (Interview P-04)*.

High power distance cultural values [47] generate normative expectations of hierarchical deference that may inadvertently enable supervisory aggression. Nevertheless, these same norms coexist with deeply held principles of human dignity—suggesting that cultural context moderates, rather than eliminates, concerns about justice and respectful treatment.

#### Collectivism and harmony preservation

Collectivist cultural orientations that emphasize group cohesion and relationship preservation powerfully shaped employees’ decisions about whether to speak up or remain silent. As described in earlier sections, the concern about “creating enemies” reflects collectivist priorities around maintaining group harmony and preserving face [40]. These dynamics compound existing psychological safety deficits by layering cultural transgression onto the interpersonal risks already inherent in voice behavior.

#### Islamic values and behavioral expectations

Several participants drew on Islamic principles as moral frameworks for evaluating appropriate conduct in the workplace:

*“Islam teaches us to treat everyone with dignity*,* to be just*,* to stand against oppression. When supervisors abuse people*,* they are violating Islamic principles*,* not just company policy. This should matter in a Saudi organization.” (Interview P-07)*.

Islamic values centered on dignity (karama), justice (adl), and compassionate leadership [7] provide a normative architecture that should preclude mistreatment. The invocation of religious principles by participants suggests that employees draw on moral authority beyond organizational policy to frame and condemn unacceptable conduct.

#### Generational divides

Several participants identified generational differences in expectations and attitudes toward workplace conduct. These dynamics may reflect broader cultural shifts as younger employees, socialized in a context of organizational modernization, bring different expectations regarding participative management, upward voice, and the right to dignified treatment [89].

### Conceptual framework: the justice-safety vicious cycle model

**Fig. 1:**
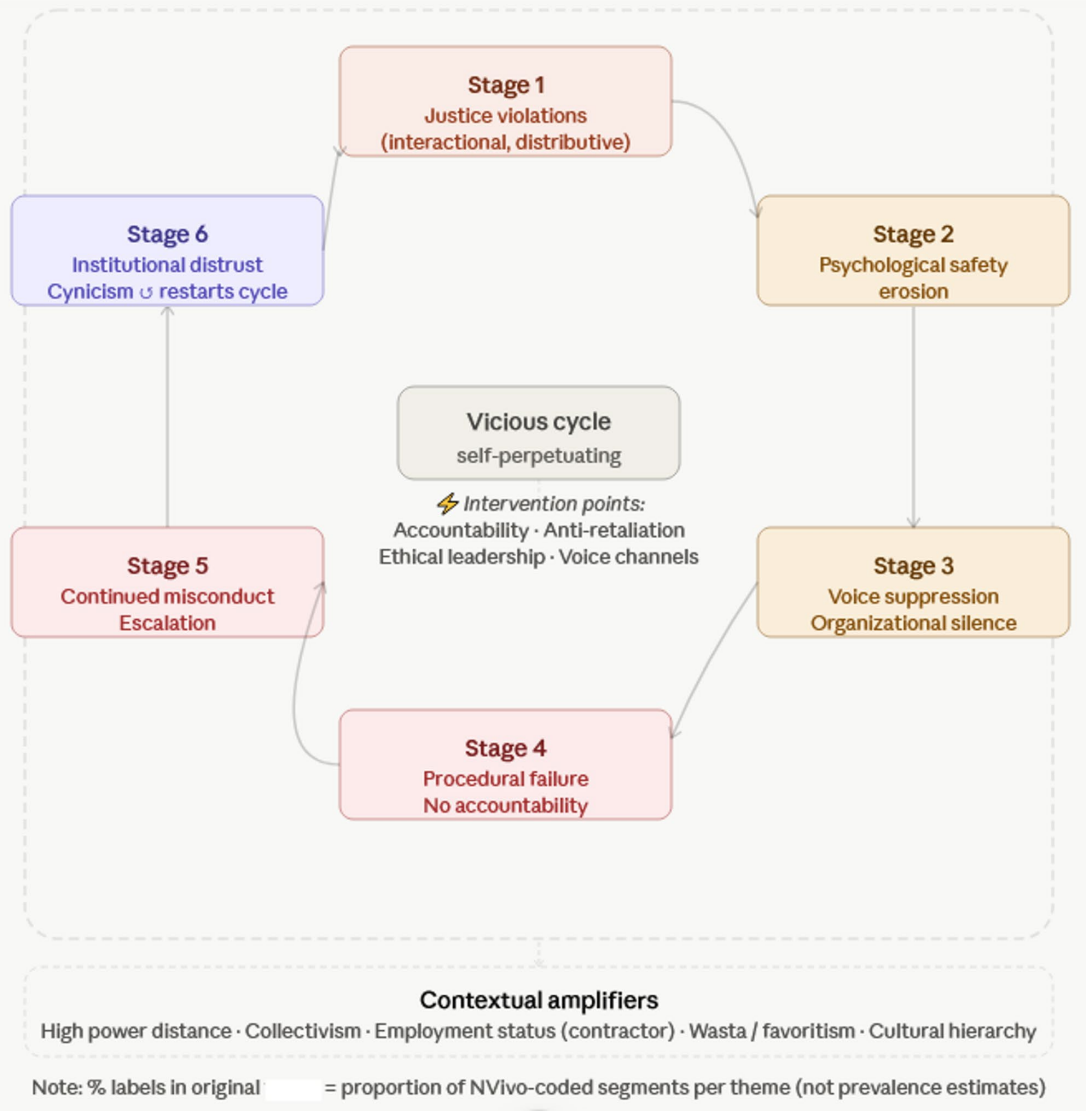
Integrated conceptual framework – the justice-safety vicious cycle model (interpretive framework grounded in participants’ accounts)

This study introduces the Justice–Safety Vicious Cycle Model (Fig. 1), an interpretive conceptual framework grounded in participants’ accounts rather than a tested causal model. It maps how unacceptable workplace behavior may perpetuate itself through six mutually reinforcing stages as described by participants. The cycle is initiated by justice violations—such as favoritism or verbal aggression—that participants reported undermined psychological safety, suppressed employee voice, and produced organizational silence. This silence, in turn, was perceived as enabling the continuation of misconduct, generating institutional distrust and cynicism. Contextual factors—cultural hierarchies, collectivist values, and employment status—function as amplifiers. The model identifies intervention points at which accountability mechanisms, ethical leadership, and anti-retaliation systems might disrupt the cycle.

The percentages in Fig. 1 reflect the proportion of NVivo-coded segments assigned to each theme and are not population-level prevalence estimates.

## Discussion

This investigation explored workers’ experiences of unacceptable workplace conduct through a theoretical lens that combined the Organizational Justice and Psychological Safety frameworks. The findings illuminate complex, self-sustaining dynamics in which justice violations erode psychological safety, suppressed voice perpetuates injustice, and systemic factors enable these patterns to persist over time.

### Alignment and extension of organizational justice theory

The findings offer strong empirical support for Organizational Justice Theory and suggest important theoretical extensions. Consistent with Bies and Moag [11], the interactional dimensions of treatment—particularly supervisory verbal aggression and public humiliation—were the most salient sources of injustice for employees. These violations elicited strong negative emotional reactions and behavioral consequences, consistent with established empirical relationships between justice perceptions and outcomes [23]. The data also confirm that interactional injustices inflicted by supervisors are particularly harmful, consistent with the “target similarity effect” [76].

Beyond these confirmatory findings, the research advances theory by demonstrating how distinct justice dimensions interact and compound one another in real organizational contexts. Whereas existing research has often examined these dimensions in isolation [22], the present data reveal that employees encounter simultaneous and interacting injustices across dimensions—with interactional violations (verbal abuse), distributive violations (perpetrators rewarded, victims penalized), and procedural violations (biased complaint handling) combining to produce pervasive and compounded fairness crises. This layering has important theoretical implications, suggesting that future justice research should prioritize examining interactive effects across dimensions rather than focusing exclusively on their independent contributions.

Novel Contribution: A previously underexplored connection emerged between procedural justice mechanisms and the infrastructure of psychological safety. Fair procedures conforming to Leventhal’s [53] criteria—characterized by consistency, bias suppression, accuracy, correctability, inclusivity, and ethical grounding—do not merely produce direct fairness perceptions. They function as signals about whether the organizational environment safely accommodates voice. When complaint processes operate inconsistently, with apparent bias, or fail to protect those who raise concerns from retaliation, they convey that speaking up is both futile and hazardous. This constitutes a meaningful theoretical extension: procedural justice systems serve as critical infrastructure for psychological safety. Organizations, therefore, cannot treat procedural justice and psychological safety as independent constructs—they are fundamentally intertwined.

Regarding cross-cultural applicability, the findings contribute to ongoing theoretical debates. A subset of participants acknowledged that cultural acceptance of hierarchical authority might render certain supervisory behaviors more normalized, consistent with arguments that high-power-distance cultures afford supervisors greater behavioral latitude [17]. However, the substantial majority of participants condemned abusive conduct regardless of their cultural background, citing universal dignity principles and Islamic values. This aligns with the view that fundamental justice principles transcend cultural boundaries [52] while recognizing that culture shapes the forms, thresholds, and responses to violations. The findings demonstrate that organizational justice theory retains relevance in non-Western settings but requires culturally sensitive application.

### Alignment and extension of psychological safety theory

The findings illustrate the centrality of psychological safety to understanding how employees respond to workplace mistreatment, consistent with the theoretical frameworks of Edmondson [32] and Morrison and Milliken [63]. As predicted, employees suppressed their voices not out of indifference but as a rational response to anticipated negative consequences. The specific deterrents identified—fear of career retaliation, concerns about relational damage, anticipated futility, and institutional distrust—align precisely with established mechanisms of voice suppression in the literature [60, 62].

Novel Contribution: The research identifies the weaponization of performance management as a particularly insidious mechanism for destroying psychological safety. Because performance ratings exert decisive influence over bonuses, promotions, development opportunities, and continued employment, supervisors who control these ratings wield extraordinary power to punish voice. This finding extends theory by revealing how organizational systems, when stripped of adequate procedural safeguards, can themselves destroy psychological safety—transforming ostensibly objective evaluation instruments into tools of coercion. It follows that psychological safety research must attend not only to interpersonal dynamics such as leader behavior and team norms, but also to the organizational systems that either enable or constrain voice.

The study supports Morrison and Milliken’s [63] distinction between individual-level silence and systemic organizational silence. The data reveal not merely isolated cases of employees fearing to speak up, but rather widespread, shared beliefs that voicing concerns causes harm rather than benefit. Multiple participants described witnessing colleagues being punished for reporting misconduct, and these vicarious experiences profoundly shaped collective perceptions of safety through social learning processes [6]. This finding resonates with recent scholarship emphasizing climate-level psychological safety [68]. It suggests that effective interventions must target systemic beliefs and organizational climates rather than focusing solely on individual-level fears.

While Edmondson [34] emphasizes leaders’ role in cultivating psychological safety, the present findings surface a darker corollary: leadership behavior can actively dismantle it. When supervisors issue explicit threats, publicly demean employees, or retaliate against those who raise concerns, they create organizational climates of fear that are fundamentally antithetical to psychological safety. This extends theory by highlighting the need for frameworks to address not only safety-building behaviors—such as accessibility and non-defensive responsiveness—but also safety-destroying behaviors, including intimidation, retaliation, and public humiliation. This bidirectional framing carries important theoretical and practical implications.

The findings further illuminate how cultural values and organizational structures amplify barriers to psychological safety. High power distance norms heighten apprehension about challenging authority; collectivist orientations compound concerns about damaging relationships; and the vulnerabilities associated with contractor status intensify fears about career consequences. These contextual factors do not simply add to existing psychological safety deficits—they interact with and multiply them. This finding calls for greater cultural contextualization within psychological safety theory, consistent with cross-cultural organizational behavior scholarship [40].

### The Justice-safety vicious cycle: theoretical integration and novel contribution

The most substantive theoretical contribution of this study lies in demonstrating how justice violations and psychological safety deficits interact in self-perpetuating vicious cycles. This cyclical model represents a novel theoretical integration that extends both justice and psychological safety frameworks.

The model proposes six recursive stages: (1) initial justice violations involving supervisory misconduct constitute interactional and distributive injustice; (2) these violations signal that the environment fails to protect dignity, eroding psychological safety; (3) diminished psychological safety suppresses the voice behaviors needed to report misconduct; (4) in the absence of voice, formal complaint mechanisms are never activated, generating procedural justice failures; (5) without accountability, perpetrators continue and may even escalate their misconduct, perpetuating injustice; and (6) the continuation of unchecked injustice further erodes psychological safety and institutional trust. The cycle then repeats, with each iteration deepening both justice deficits and the erosion of safety.

This model contributes to theory in several ways. First, it positions justice and safety not merely as correlated constructs but as mutually constitutive and dynamically interdependent phenomena that cannot be effectively addressed in isolation. Second, it reveals the temporal sequencing of these dynamics: justice violations precede the erosion of safety, which in turn precedes the suppression of voice, which in turn perpetuates further injustice—a recursive pattern with significant implications for intervention timing and design. Third, the model accounts for its own self-perpetuation: each stage generates the conditions that enable the next, creating momentum that is difficult to disrupt without multilevel intervention.

While prior scholarship has recognized connections between justice and psychological safety [19, 80], this study provides detailed empirical grounding for the reciprocal dynamics at work in organizational mistreatment contexts. The vicious cycle model offers a theoretical framework for understanding why workplace mistreatment proves so resistant to superficial remedies and why sustained, multilevel approaches are necessary for meaningful change.

### Novel empirical contributions

In addition to its theoretical extensions, the research identifies several empirical phenomena that have received limited prior attention in the literature:

Performance Management Weaponization: While existing research has documented political bias in performance appraisal processes [27], this study uncovers a more deliberate and systematic form of weaponization—whereby supervisors explicitly manipulate performance ratings to punish those who speak up, enforce compliance, and retaliate against formal complaints. This confronts employees with an untenable choice: accept mistreatment and preserve career prospects, or report it and suffer consequences. The finding has direct implications for how performance management systems should be designed and safeguarded.

Employment Status Intersectional Vulnerabilities: The research reveals how workers’ employment classifications create differentiated vulnerabilities to mistreatment. Contractors reported pervasive disrespect, elevated exposure to abusive supervisory conduct, exclusion from organizational protections, and compounded power asymmetries. This pattern reflects intersectional vulnerability—not merely the accumulation but the multiplication of risks at the intersection of employment status, power imbalance, and constrained voice. This extends contingent worker research [54] by demonstrating how employment classification interacts dynamically with justice and safety dynamics. Critically, employment status-based discrimination represents a structural injustice at the organizational level that demands systemic rather than individualized remediation.

Policy-Practice Gaps and Cynicism: While psychological contract breach has been extensively theorized [75], this study sheds light on the particular nature of the gaps that generate cynicism in this context. Organizations that loudly proclaim ethical commitments while tacitly permitting violations create an especially corrosive dynamic: employees perceive institutional hypocrisy, which proves more damaging to trust than the absence of commitments altogether [79]. The finding that “zero tolerance” policies go unenforced—while perpetrators are shielded and victims face retaliation—illustrates how organizations can inadvertently undermine employee trust and engagement through the very policies designed to promote them.

### Contextual dynamics: cultural factors and universal principles

The research contributes to scholarly understanding of workplace behavior in Middle Eastern organizational contexts while revealing productive tensions between contextual particularities and universal principles. Context-specific features included high power distance norms that generated stronger expectations of hierarchical deference; collectivist orientations that amplified concerns about relationship damage; Islamic principles that furnished normative frameworks condemning mistreatment; and intricate intersections of tribal, familial, and national status that produced layered power dynamics.

Nevertheless, universal principles emerged with equal clarity: fundamental needs for dignity and respect transcended cultural backgrounds; violations of justice elicited negative reactions across nationalities; employees from diverse contexts converged in condemning abusive conduct; and the dynamics of psychological safety operated consistently across groups. Taken together, these findings suggest that while cultural context shapes the forms, thresholds, and response strategies associated with mistreatment, core principles of justice and psychological safety retain relevance across cultural boundaries.

This finding carries important theoretical and practical implications. It demonstrates that organizations operating in non-Western contexts cannot dismiss employee concerns about mistreatment as culturally inappropriate impositions from Western frameworks. Workers universally value dignity and fairness. At the same time, cultural sensitivity remains essential—and culture cannot be invoked as justification for tolerating abuse. This position aligns with recent cross-cultural organizational behavior scholarship emphasizing the interplay between universal principles and cultural contingencies [41].

### Comparison with existing literature

Alignment: The findings converge with an extensive body of literature documenting the prevalence of workplace mistreatment [24, 70]; its harmful effects on victims including psychological distress and performance decrements [12, 46]; the centrality of power asymmetries to mistreatment dynamics [85]; fear of retaliation as the primary barrier to voice [65]; and beliefs about futility as a reinforcing mechanism for organizational silence [62].

Extension: However, the study advances existing knowledge in several ways. While prior research has documented the prevalence of mistreatment primarily through survey methods, this investigation provides rich qualitative insight into the lived experiences and interpretive processes of those affected. Where existing research has identified barriers to voice, this study exposes the precise mechanisms through which performance management systems are weaponized as instruments of coercion. Furthermore, where prior research has acknowledged cultural influences in general terms, this study demonstrates how cultural factors interact with and amplify justice and safety dynamics through multiplicative rather than merely additive effects.

Divergence: The findings diverge from perspectives suggesting that high power distance cultures normalize supervisory aggression [92]. While some participants acknowledged cultural respect for authority, the overwhelming majority condemned abusive conduct, suggesting that dignity violations remain widely experienced as problematic even within hierarchical cultural contexts. This is consistent with more recent perspectives that affirm universal justice needs while acknowledging variation in cultural expressions of those needs [17, 52].

## Research contributions, limitations, and future research

### Theoretical contributions

#### Extension of organizational justice theory

First, the research demonstrates how distinct justice dimensions interact and compound in actual organizational contexts. While the majority of justice research has examined dimensions separately [21], this investigation reveals that employees simultaneously experience interactional, distributive, and procedural injustices whose combined effects multiply harm through layered, interactive processes. This points to the need for future research to adopt configurational and interactive approaches to studying justice profiles rather than examining isolated dimensions.

Second, the research establishes procedural justice systems as foundational infrastructure for psychological safety. This connection extends theory by demonstrating that procedural fairness mechanisms fulfill a dual function: shaping perceptions of outcome fairness and signaling whether the organizational environment safely accommodates voice. Organizations that treat procedural justice design and the cultivation of psychological safety as separate initiatives fail to recognize their fundamental interdependence. This integration enriches both theoretical traditions.

Third, by examining justice dynamics in an underrepresented, non-Western, high-power-distance, and collectivist setting, the research contributes to the cross-cultural justice literature. The findings affirm the universal relevance of justice principles while demonstrating that cultural context moderates their expression and the strategies employees use to respond to violations—advancing a more nuanced understanding of the scope conditions of justice theory.

Fourth, the research illustrates how structural arrangements—particularly employment-status hierarchies—produce systemic violations of justice. This extends justice theory beyond interpersonal interactions to encompass organizational-level structural inequities, suggesting that justice scholars should attend to policy-embedded disparities alongside individual-level perceptions of fairness.

Fifth, the findings demonstrate how justice violations generate not merely individual-level reactions but collective cynicism and institutional distrust, advancing understanding of organizational-level justice climate dynamics [2].

#### Extension of psychological safety theory

First, the research establishes a reciprocal relationship between justice violations and the erosion of safety, positioning them as mutually constitutive phenomena that interact dynamically rather than as independent variables. This integration enriches both literatures by making visible the bidirectional mechanisms linking them.

Second, the study identifies specific mechanisms by which supervisory behavior destroys psychological safety—including intimidation, public humiliation, and retaliation—thereby extending theoretical frameworks beyond safety-building to encompass safety-destroying dynamics. This bidirectional perspective strengthens both theoretical understanding and practical guidance.

Third, the research reveals how organizational systems, such as performance management, can undermine psychological safety when exploited for retaliatory purposes. This extends theory beyond interpersonal dynamics to encompass organizational systems that require structural safeguards to prevent misuse.

Fourth, by examining how cultural factors such as power distance and collectivism amplify psychological safety barriers through interactive rather than merely additive effects, the research underscores the importance of cultural contextualization within psychological safety theory and advances cross-cultural organizational behavior scholarship.

Fifth, the study provides rich empirical grounding for the emergence of systemic organizational silence through vicarious learning and the formation of collective beliefs, thereby extending Morrison and Milliken’s [63] foundational framework.

#### Theoretical Integration: the vicious cycle model

The most significant theoretical contribution of this study is the introduction of the Justice-Safety Vicious Cycle Model, which explains the self-sustaining dynamics underlying workplace mistreatment. This model advances theory by:

- Positioning justice and psychological safety as mutually constitutive rather than merely correlated constructs.
- Mapping the temporal sequence: justice violations → safety erosion → voice suppression → procedural failures → continued injustice → further safety collapse.
- Explaining the self-perpetuating mechanisms that generate momentum, making it difficult to interrupt.
- Identifying multiple intervention points that require simultaneous attention.
- Providing an integrative framework that synthesizes previously separate theoretical traditions.

This cyclical model offers a theoretical lens for understanding why workplace mistreatment tends to resist superficial remedies, and why sustained, multilevel approaches to intervention are essential. The model generates testable propositions for future quantitative research and provides actionable direction for practitioners engaged in organizational change.

### Empirical contributions

#### Rich contextualized understanding

This study offers detailed, nuanced, and theoretically grounded insight into how employees in a large Saudi multinational organization experience, make sense of, and respond to unacceptable workplace conduct. In doing so, it addresses the severe underrepresentation of Middle Eastern contexts in organizational behavior scholarship [59], contributing an empirically anchored understanding of workplace dynamics in Saudi Arabia and illuminating both the universal and culturally specific dimensions of these experiences.

#### Employee voice captured

By integrating analysis of organically generated employee discourse from the internal social media with in-depth interview data, the study captures authentic organizational conversations and employee-defined priorities rather than imposing researcher-constructed frameworks. This methodological approach provides access to employees’ spontaneous, self-initiated expressions of concern, revealing what they themselves find most salient and troubling.

#### Identification of novel phenomena

The research surfaces phenomena that have received limited scholarly attention:

- Performance management weaponization as a deliberate and systematic mechanism of retaliation.
- Employment status-based discrimination creates intersectional vulnerabilities.
- Specific policy-practice gaps that generate cynicism, including the non-enforcement of zero tolerance commitments and the protection of perpetrators while victims face retaliation.
- Cultural–structural interactions that amplify psychological safety barriers through multiplicative rather than additive effects.

These novel findings open productive avenues for future research and inform the design of practical interventions.

### Methodological contributions

#### Dual-source triangulation

The integration of content analysis of internal social media comments with semi-structured interviews represents methodological innovation, combining breadth—222 comments drawn from a diverse population—with depth—39 detailed interview accounts. This approach addresses the limitations inherent in single-method designs. Comments from the internal social media provide naturally occurring data that are less susceptible to social desirability effects, while interviews enable probing and clarification of complex experiences. The convergence of findings across sources substantially enhances the credibility of the results.

#### Naturally occurring data

Analysis of employee-generated content from the organization’s internal social media provides access to authentic organizational discourse that captures how employees frame their concerns when communicating within their own professional community. This source offers advantages over researcher-elicited responses by reflecting spontaneous expressions unmediated by interviewer influence.

#### Theory-driven qualitative analysis

The systematic application of established theoretical frameworks—Organizational Justice and Psychological Safety—to qualitative data demonstrates how theory-driven analysis can generate both descriptive richness and analytical depth. This approach bridges the divide between purely exploratory qualitative inquiry and theory-testing quantitative research, advancing the methodological sophistication of qualitative organizational scholarship.

### Practical contributions

The research provides evidence-based guidance for organizational practitioners:

The following recommendations map directly to intervention points identified in the Justice–Safety Vicious Cycle Model (Fig. 1), each targeting a specific stage at which the self-perpetuating dynamic can be disrupted. Establish Real Accountability (Breaking Stages 4–5—Procedural Failure and Continued Misconduct): Organizations should implement transparent and consistent procedures for investigating misconduct; ensure that investigators are genuinely impartial and free from conflicts of interest; apply policies equitably across all organizational levels; enforce stated consequences so that “zero tolerance” commitments carry real meaning; incorporate subordinate feedback through 360-degree evaluation systems; and establish external oversight functions independent of existing management hierarchies.

- Protect and Promote Voice: Organizations should establish multiple, accessible voice channels, including anonymous reporting mechanisms and ombudspersons; guarantee credible confidentiality protections with explicit and visible anti-retaliation safeguards; communicate consistently that employee concerns are valued and will be acted upon; demonstrate responsiveness by ensuring that concerns actually prompt action; and equip managers with the skills and dispositions needed to receive upward feedback non-defensively.
- Address Systemic Justice Issues: Organizations should eliminate employment status-based differentiation by extending dignity and protection to all workers; counter favoritism through transparent, merit-based personnel processes; ensure procedural consistency in the application of policies across organizational levels; and conduct regular audits of perceptions of justice, disaggregated by relevant employee characteristics.
- Leadership Selection and Development: Organizations should revise criteria for supervisory selection to assess interpersonal competence and ethical leadership capacity; require leadership development programming centered on respectful management, psychological safety cultivation, and justice principles; introduce probationary arrangements for newly appointed supervisors with explicit behavioral expectations; and remove individuals who demonstrate a persistent inability to lead respectfully.
- Performance Management Reform: Organizations should establish procedural safeguards to prevent performance ratings from being manipulated for retaliatory purposes; implement calibration processes that involve multiple evaluators; provide clear appeal mechanisms for employees who contest their ratings; and structurally separate performance evaluation processes from misconduct complaint investigation procedures.
- Psychological Contract Management: Organizations should ensure that espoused values are consistently enacted in practice; avoid making commitments that cannot be reliably honored; periodically assess and publicly report on progress toward ethical climate objectives; and acknowledge shortfalls while demonstrating genuine commitment to continuous improvement.

### Contextual contributions

This research directly addresses the marked underrepresentation of Middle Eastern contexts in the organizational behavior literature, contributing an empirically grounded understanding of workplace dynamics in Saudi Arabia. The findings are relevant to organizations navigating the tension between modernization pressures and the preservation of cultural values—balancing traditional hierarchical structures with contemporary expectations for dignity, participative management, and employee voice. Given the extreme workforce diversity of the organization studied, the findings also illuminate the challenges confronting multinational organizations that manage employees from vastly different cultural backgrounds, pointing to the need for explicit behavioral norms that can transcend cultural particularities.

### Limitations

Despite its contributions, the study exhibits several limitations that warrant acknowledgment. First, as a single-case investigation, its findings may not generalize to other organizations, sectors, or national settings. Although the organization shares characteristics with other large Middle Eastern multinationals—including scale, workforce diversity, hierarchical structure, and modernization tensions—each organizational context possesses unique features. The theoretical propositions generated here require testing across multiple cases.

Second, the temporal gap between data sources—social media comments from 2014 and interviews from 2017–2020—warrants acknowledgment. While this gap was treated analytically as an opportunity for cross-temporal comparison, organizational changes during the intervening period may have affected participants’ accounts in ways difficult to disaggregate. Readers should interpret cross-source comparisons with appropriate caution. Third, self-selection effects apply to both data sources. Individuals who participated in the content analysis by posting comments and those who agreed to be interviewed both chose to do so. Those who had experienced or observed mistreatment may have been more motivated to participate, potentially overrepresenting negative experiences—a negativity bias inherent in self-selected discourse data [51]. Nonetheless, the number of comments (222) and the deliberate diversity of interview participants’ profiles suggest a reasonably comprehensive representation.

Fourth, social desirability effects may have influenced how participants presented their experiences despite assurances of confidentiality. Employees may have moderated their most negative views when communicating in organizational channels—even anonymously—particularly within hierarchical cultural contexts where criticism of authority can be perceived as inappropriate. The power dynamics inherent in interviews—where employees may perceive researchers as affiliated with management—may also have constrained authentic expression. Methodological triangulation and the availability of anonymous written comments partially mitigate this limitation, but social desirability effects cannot be eliminated.

Fifth, data collection occurred during defined time windows (2017–2020), capturing perspectives from that specific period. Longitudinal research designs would more effectively illuminate how experiences, perceptions, and organizational responses evolve over time and whether vicious cycles can be interrupted through targeted interventions.

### Future research directions

The findings and limitations identified above suggest several productive directions for future inquiry. First, comparative research should examine workplace behavior dynamics across: (1) multiple Saudi organizations varying in sector, size, and ownership structure; (2) Middle Eastern countries with differing cultural characteristics and governance frameworks; (3) Middle Eastern and Western organizational contexts, systematically testing the cross-cultural applicability of theoretical propositions; and (4) organizations with varying workforce compositions to examine how diversity affects justice and safety dynamics.

Second, longitudinal research designs would enable investigation of: (1) how patterns of workplace conduct, justice perceptions, and psychological safety evolve; (2) organizational trajectories following specific interventions, testing whether vicious cycles can be broken; (3) career consequences of voice versus silence over extended timeframes; and (4) the long-term effects of mistreatment on individual well-being and career development.

Third, action research or quasi-experimental designs could evaluate the effectiveness of specific interventions: (1) comparing alternative complaint system architectures; (2) assessing the impact of leadership development initiatives on supervisory conduct and psychological safety; (3) evaluating the effectiveness of accountability mechanisms in reducing mistreatment and encouraging voice; and (4) examining outcomes of culturally sensitive organizational training programs.

Fourth, multi-level research designs examining individual, team, and organizational levels simultaneously would better capture the nested dynamics that single-level approaches inevitably miss. Hierarchical linear modeling could illuminate how individual differences, team climates, and organizational cultures interact to shape the incidence and experience of unacceptable conduct—enabling investigation of cross-level effects such as whether organizational-level practices affect individuals differently depending on personality or role.

Fifth, future research should incorporate broader stakeholder perspectives by including: (1) the viewpoints of supervisors and managers regarding the challenges and constraints they face; (2) HR professionals’ perspectives on the challenges of system design and implementation; (3) executive leadership accounts of cultural change efforts and organizational barriers; and (4) comparative analyses of the experiences and interpretations of perpetrators, victims, and bystanders.

Sixth, further theoretical development is needed to explore: (1) the boundary conditions of the Justice-Safety Vicious Cycle Model—specifically, under what circumstances cycles break or reverse; (2) the mechanisms through which virtuous cycles emerge and sustain themselves; (3) the potential role of organizational trust as a mediator or moderator; and (4) integration of the present framework with complementary theoretical perspectives, including social identity theory, conservation of resources theory, and psychological contract theory.

## Data Availability

No datasets were generated or analysed during the current study.
